# Unveiling the multifaceted roles of microRNAs in extracellular vesicles derived from mesenchymal stem cells: implications in tumor progression and therapeutic interventions

**DOI:** 10.3389/fphar.2024.1438177

**Published:** 2024-08-05

**Authors:** Sujia Hu, Chang Zhang, Qianhui Ma, Minghe Li, Xiao Yu, Haiying Zhang, Shuang Lv, Yingai Shi, Xu He

**Affiliations:** The Key Laboratory of Pathobiology, Ministry of Education, College of Basic Medical Sciences, Jilin University, Changchun, Jilin, China

**Keywords:** mesenchymal stem cells, extracellular vesicles, microRNAs, tumor progression, tumor therapeutic applications

## Abstract

Mesenchymal stem/stromal cells (MSCs) have the capacity to migrate to tumor sites *in vivo* and transmit paracrine signals by secreting extracellular vesicles (EVs) to regulate tumor biological behaviors. MSC-derived EVs (MSC-EVs) have similar tumor tropism and pro- or anti-tumorigenesis as their parental cells and exhibit superior properties in drug delivery. MSC-EVs can transfer microRNAs (miRNAs) to tumor cells, thereby manipulating multiple key cancer-related pathways, and further playing a vital role in the tumor growth, metastasis, drug resistance and other aspects. In addition, tumor cells can also influence the behaviors of MSCs in the tumor microenvironment (TME), orchestrating this regulatory process via miRNAs in EVs (EV-miRNAs). Clarifying the specific mechanism by which MSC-derived EV-miRNAs regulate tumor progression, as well as investigating the roles of EV-miRNAs in the TME will contribute to their applications in tumor pharmacotherapy. This article mainly reviews the multifaceted roles and mechanism of miRNAs in MSC-EVs affecting tumor progression, the crosstalk between MSCs and tumor cells caused by EV-miRNAs in the TME. Eventually, the clinical applications of miRNAs in MSC-EVs in tumor therapeutics are illustrated.

## Graphical Abstract

**Figure:**
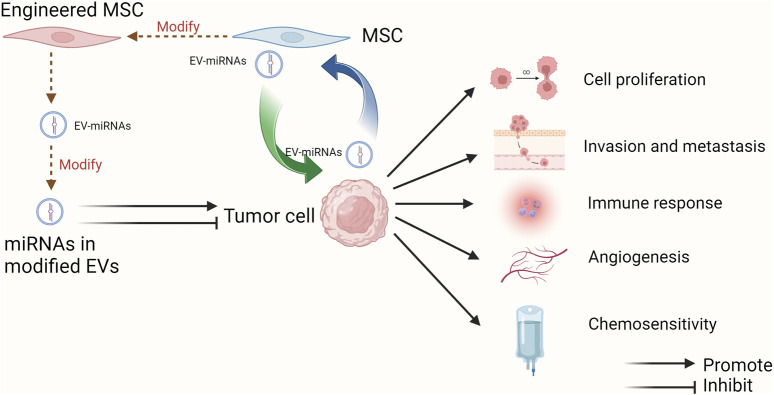
The multifaceted roles of EV-miRNAs derived from MSCs in tumor progression and therapeutic applications. MSC-EVs can transfer miRNAs into tumor cells to regulate cell proliferation, invasion and metastasis, immune response, angiogenesis, and chemosensitivity as well. One or more of MSCs, EVs and miRNAs can be modified to achieve the inhibitory effect on tumor progression, which provides the promising approaches for tumor clinical treatment. Besides, miRNAs in EVs can also mediate the interaction between MSCs and tumor cells in the TME (Created with Biorender.com).

## 1 Introduction

**FIGURE 1:**
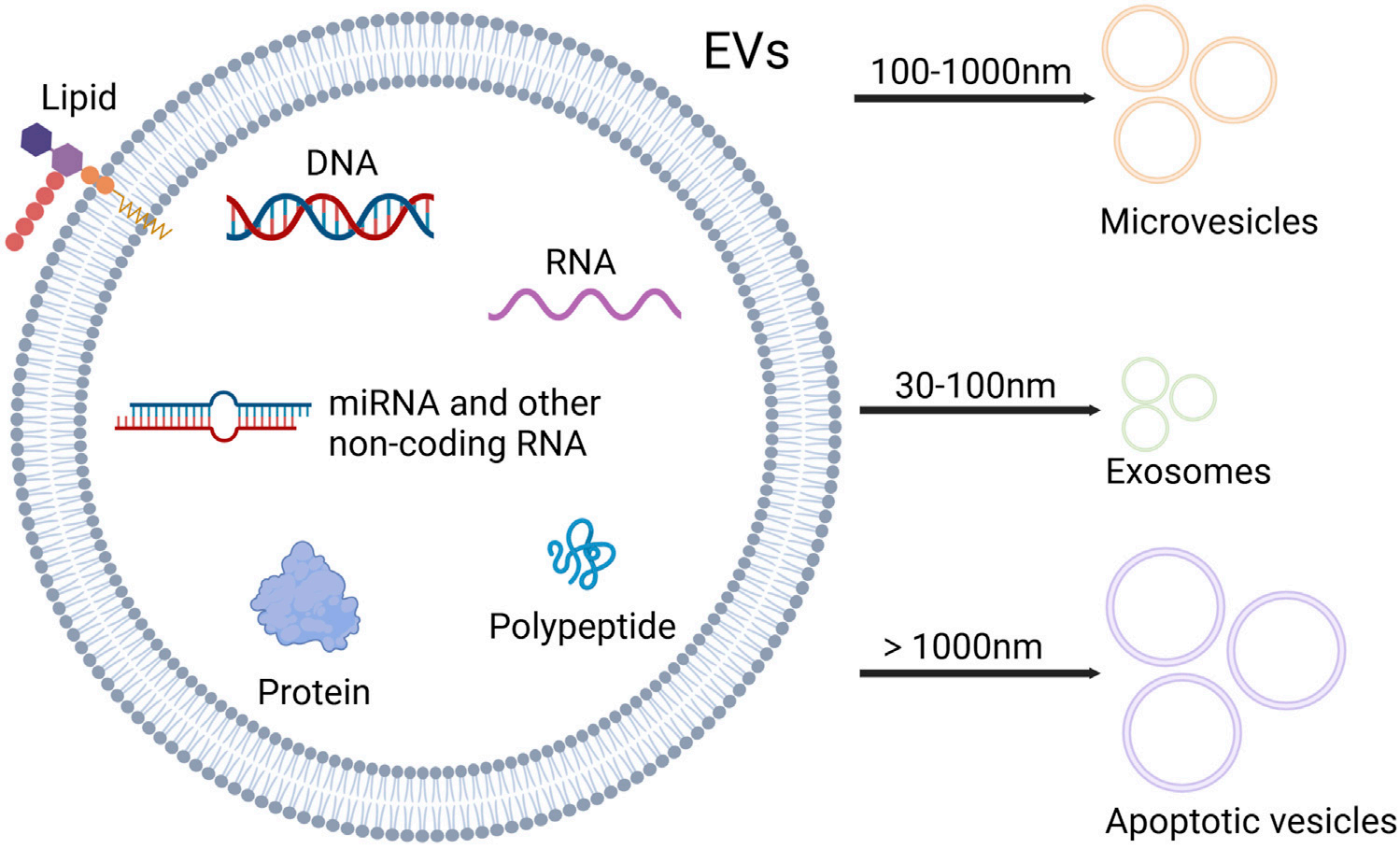
Contents and types of EVs. EVs are rich in nucleic acids, proteins and lipids. Based on the size, EVs can be categorized into three types: microvesicles, exosomes and apoptotic vesicles. Microvesicles range from 100 to 1,000 nm in diameter, and diameter of exosomes varies between 30 and 100 nm, while apoptotic vesicles exceed 1,000 nm in diameter (Created with Biorender.com).

Mesenchymal stem/stromal cells (MSCs) are a group of non-hematopoietic multipotent stem cells with the ability of self-renewal and multi-lineage differentiation, which can participate in tissue regeneration and homeostasis, and have immunomodulatory properties (Ullah et al., 2015; Wang Y. et al., 2022). MSCs can be isolated from a variety of human tissues, including bone marrow, umbilical cord, adipose tissue, skin, dental pulp, endometrium, and so on. Bone marrow is the most important source of MSCs (Dabrowska et al., 2021). MSCs can influence the surrounding cells through paracrine effects, and their secretory components consist of various bioactive factors including extracellular vesicles (EVs) (Harrell et al., 2019; Mitchell et al., 2019). EVs are the nano-sized vesicles secreted by cells, which contain proteins, lipids, nucleic acids, metabolites, growth factors, and cytokines, playing an essential role in mediating cellular communication (Alzahrani et al., 2024; Cabrera-Pastor, 2024). Based on the size and biogenesis, EVs can be categorized into exosomes, microvesicles (MVs) and apoptotic vesicles (Zhang F. et al., 2022) (Figure 1). MicroRNAs (miRNAs), as an important component of EVs, are small non-coding RNAs of about 18–25 nucleotides in length (Yao et al., 2019). MiRNAs can be paired with mRNAs to exert the post-transcriptional inhibition by translational repression, mRNA destabilization, and mRNA cleavage. Besides, a single miRNA can target multiple different mRNAs, and a single mRNA can be coordinately suppressed by multiple different miRNAs. Thus, miRNAs have vital roles in gene regulatory networks (Bartel, 2009; Lin and Gregory, 2015).

Tumors are complex tissues, which establish a unique pathological environment, tumor microenvironment (TME), during the process of their dynamic development. TME is characterized by a hypoxic, acidic environment. The major cellular components of TME include immune cells, stromal cells, such as cancer-associated fibroblasts (CAFs), pericytes, MSCs, as well as the other non-cellular components such as extracellular matrix (ECM), vascular network and lymphatic system, and multiple signaling molecules (Feng et al., 2023; Zhang et al., 2023). In this environment, the interplay between stromal and malignant cells critically determines tumor progression (Terry et al., 2017).

In recent years, there has been increasing evidence that miRNAs carried by MSC-derived EVs (MSC-EVs) play a crucial role in the regulation of tumor progression. Figuring out the complex impacts of miRNAs in MSC-EVs on tumors will provide the novel ideas for tumor diagnosis, treatment and monitoring. This review first summarized the roles and mechanism of miRNAs in MSC-EVs that influence tumor progression, and then described the crosstalk action of miRNAs in EVs between tumor cells and MSCs. Finally, the application prospects of miRNAs in MSC-EVs for tumor clinical therapeutics was explored.

## 2 Isolation of EVs

The extraction of EVs is a prerequisite for biomedical research and clinical translation (Li et al., 2024). Different isolation methods can significantly affect the cargo and physicochemical properties of EVs (De Sousa et al., 2023). Currently, a multitude of separation techniques have been developed.

Ultracentrifugation is considered as the “golden standard” technique for the separation of EVs (Gardiner et al., 2016), mainly comprising differential ultracentrifugation, density gradient ultracentrifugation (Li et al., 2024). As the most commonly used method, ultracentrifugation has the characteristics of mature technology, wide applicability and low cost. However, the drawbacks of time-consuming, poor reproducibility, large sample volume required and high impurities also limit its applications (Lucchetti et al., 2019; Chen et al., 2022).

Size exclusion chromatography uses polymers to form a porous stationary phase in a column to separate EVs (Jia et al., 2022). Although short in time, low in cost, and high in purity, it requires special columns and packings and is prone to lipoprotein contamination (Böing et al., 2014; Chen et al., 2022).

Immunoaffinity capture isolates EVs by binding to specific membrane receptors on the surface of EVs and specific antibodies to obtain high-purity EVs, but it is low- yield and expensive (Alzhrani et al., 2021; Stam et al., 2021).

The method of precipitation separates EVs from other compounds based on the solubility, and polyethylene glycol is often used to precipitate EVs (Coumans et al., 2017; Soares Martins et al., 2018). Precipitation is easy to operate, inexpensive, and suitable for large sample size (Chen et al., 2022).

In addition to the above methods, ultrafiltration can concentrate EVs into a small volume sample for EV separation (Zhu et al., 2020). Microfluidics relies on the physical and biochemical properties to isolate EVs (Alzhrani et al., 2021). In recent years, separation methods of EVs have been further developed via utilizing advanced materials (Yang et al., 2021; Dao et al., 2022; Kim et al., 2024) and techniques (Chen et al., 2021).

## 3 Roles and mechanism of miRNAs in MSC-EVs in tumor progression

MSCs are multipotent stem cells, which can be recruited into the TME to become tumor-associated MSCs (Timaner et al., 2020). Tumor-associated MSCs can influence tumor progression by transmitting paracrine signals through the secretion of EVs. Currently, MSCs are widely used in cancer research because of their ability to migrate to the tumor site after systemic administration and interact with tumor cells (Belmar-Lopez et al., 2013; Alzahrani et al., 2018) and their ease of isolation as well as *ex vivo* culture and modification. In addition, MSC-EVs can mimic their parental cells, migrate to the tumor sites, and exert an impact on them (Weng et al., 2021). Due to diverse tumor types, stem cell sources, isolation methods, and *in vitro* culture conditions, MSC-EVs have two opposite roles in tumor progression: promotion and inhibition (Belmar-Lopez et al., 2013; Yassine and Alaaeddine, 2022; Aldoghachi et al., 2023).

As early as 2014, it was demonstrated that paclitaxel (PTX)-treated MSCs could secrete PTX-containing MVs, possessing the significant anti-tumor effects. This finding suggested the potential applications of MSC-EVs for drug packaging and delivery. Furthermore, the pharmacological activity of PTX was not affected during the physiological biogenesis of MVs (Pascucci et al., 2014). More importantly, EVs obtained the better therapeutic efficacy with more limited side effects than free drugs (Namee and O’Driscoll, 2018). Another study compared the delivery efficiency of modified exosomes derived from transgenic dental pulp MSCs and liposomes for the delivery of anti-tumor miRNAs to breast cancer cells, demonstrating the promising applications of exosomes derived from MSCs in drug delivery (Vakhshiteh et al., 2021).

In different types of cancer cells, miRNA expression appears to be up-regulated or down-regulated (Wang et al., 2014; Wu et al., 2020; Xing et al., 2020; Khazaei-Poul et al., 2021; Liu J. et al., 2021; Wang and Lin, 2021; Yan et al., 2021; Jiang et al., 2022; Lv et al., 2022; Wang X. et al., 2022; Wang X.-S. et al., 2022; Zhao et al., 2022; Orso et al., 2023), which correlates with tumor prognosis (Lan et al., 2018; Xing et al., 2020; Liu J. et al., 2021; Wang X. et al., 2022), tumor grading (Xing et al., 2020), TNM staging (Wang et al., 2014; Wu et al., 2020) and differentiation degree (Wu et al., 2020). This indicates that targeting miRNAs can manipulate the tumor biological behaviors. The strategies to regulate the expression of miRNAs in tumors include the inactivation of oncogenic miRNAs, the activation of tumor suppressor miRNAs, and the restoration of drug sensitivity via targeting specific miRNAs (Sarkar et al., 2010). Moreover, the expression levels of miRNAs in EVs (EV-miRNAs) in MSCs derived from the patients with malignant tumors are different from those of normal subjects (Roccaro et al., 2013; Wang et al., 2014; Figueroa et al., 2017). The characteristics of miRNAs in MSC-EVs in tumors has been extensively studied so far, and MSCs are often modified to investigate the effects of miRNAs in modified MSC-EVs on tumor progression.

A growing number of studies have shown that miRNAs in MSC-EVs are involved in the regulation of key cancer-related pathways (Bushati and Cohen, 2007; Jansson and Lund, 2012). These pathways serve as diverse functions in tumor progression, including modulating tumor cell proliferation, invasion, metastasis and angiogenesis, dominating the host immune responses, as well as manipulating tumor chemoresistance. In addition, it has been demonstrated that miRNAs in MSC-derived exosomes (MSC-Exos) also participate in the development of precancerous lesions (Li et al., 2019; Wang et al., 2019).

### 3.1 Influence on tumor cell proliferation

Maintaining the capacity of long-term proliferation is the most fundamental characteristic of tumor cells, which can influence cell cycle progression as well as cell growth by modulating growth-promoting signals (Hanahan and Weinberg, 2011). As early as 2013, it was found that MVs derived from human bone marrow mesenchymal stem cells (BMSCs) inhibited cell cycle progression and induced apoptosis or necrosis in hepatocellular carcinoma (HCC), Kaposi’s sarcoma, and ovarian tumors (Bruno et al., 2013). Another later study discovered that adipose mesenchymal stem cell (AMSC)-derived exosomes had a similar effect on ovarian cancer (OC) cells, which was mediated by RNAs rather than proteins in the exosomes. And it was concluded in further study that miRNAs in AMSC-Exos were responsible for the above observed anti-tumor activity (Reza et al., 2016). Increasing evidence indicates that targeting cell cycle-related proteins or signaling pathways, and inducing or repressing cell apoptosis are common mechanisms, by which miRNAs in MSC-EVs orchestrate tumor cell proliferation in a variety of tumors.

#### 3.1.1 Tumor cell proliferation suppression

A case in point is the study by Xu et al. They found that BMSC-Exos inhibited cell proliferation and cycle progression and promote apoptosis in acute myeloid leukemia (AML) cells. Knock-down of miR-124-5p in BMSCs eliminated this modulatory effect, suggesting that miR-124-5p could contribute to the exosomal impacts observed in AML cells (Xu et al., 2020). By suppressing JAG1 expression, BMSC-derived exosomal miR-512-5p attenuated glioblastoma cell proliferation and induced cell cycle arrest (Yan et al., 2021). Similarly, miR-144 in BMSC-Exos could down-regulate the expression levels of CCNE1 and CCNE2, thereby regulating the cell proliferation and cell cycle progression in non-small cell lung cancer (NSCLC), and further inhibiting NSCLC progression (Liang et al., 2020). Additionally, MSC-Exos was enriched in miR-100, which could modulate the miR-100/mTOR/miR-143 axis, while mTOR and miR-143 could diminish the cell proliferation and enhance cell apoptosis in colorectal cancer (CRC) by affecting glycolysis, cell cycle progression, and other mechanisms. Interestingly, miR-143 was also enriched in MSC-Exos (Jahangiri et al., 2022).

One study reported that hBMSC-derived exosomal miR-484 inactivated the Wnt/MAPK pathway, relieved the proliferation and boosted apoptosis of pancreatic cancer cells *in vitro*, and reduced tumor size and weight *in vivo* (Lin et al., 2023). HBMSC-derived exosomal miR-124-3p diminished cMYC expression by down-regulating NFATc1 and exerted comparable effects on diffuse large B-cell lymphoma (Zhao et al., 2023). Besides, TRIM14 facilitated proliferation and restrain apoptosis of AML cells through the activation of PI3K/AKT pathway, while hMSC-Exos could reverse the effects of TRIM14 by delivering miR-23b-5p (Cheng et al., 2021). Another analogous study demonstrated that exo-miR-7-5p derived from BMSCs functioned by attenuating the phosphorylation of PI3K/AKT/mTOR signaling pathway to negatively regulate OSBPL11 (Jiang et al., 2022). Likewise, MSC-Exos carrying miR-133b could repress glioma cell proliferation by disrupting Wnt/β-catenin signaling pathway through EZH2 inhibition (Xu H. et al., 2019). Additionally, miR-3182 in human umbilical cord mesenchymal stem cells (hUCMSCs)-derived exosomes exerted the similar influence on triple-negative breast cancer (TNBC) cells (Khazaei-Poul et al., 2021).

Based on the different subpopulations of MSCs, Li et al. found that CD90^low^ ADSCs and their derived EVs significantly retarded the tumor growth in hormonal mice, which was associated with decreased tumor cell proliferation and migration as well as increased tumor cell apoptosis. More importantly, the pro-apoptotic effect of EVs on tumor cells was further enhanced after loading the oncogenic miRNA -miR-16-5p mimic into CD90^low^ ADSC-EVs by liposomal membrane fusion method (Li et al., 2020). Another study reported the findings that under hypoxic conditions, intracellular let-7f levels were up-regulated through HIF-1α pathway, and the secreted let-7f encapsulated in hMSC-Exos was also elevated. *In vitro* and *in vivo* experiments further proved that hMSC-Exos containing let-7f could be ingested by tumor cells, and thus attenuate tumor cell proliferation and invasion, and relieve tumor growth *in vivo*, demonstrating that let-7f in hMSC-Exos possessed the good anti-tumor activity. Besides, MSCs treated with inflammatory factor and chemokine SDF-1α can also play a similar role (Egea et al., 2021).

#### 3.1.2 Tumor cell proliferation promotion

Contrary to the above findings, many studies have provided evidence that miRNAs in MSC-EVs faciliate the tumor cell proliferation. For instance, the expression of miR-217 in bladder cancer cells was notably higher than that in normal human bladder cells. Furthermore, the exosomal miR-217 derived from normal human bladder MSCs boosted the proliferation and migration of bladder cancer cells and suppressed apoptosis by regulating the transcription factor YAP and its target proteins (Huang et al., 2021). A study comparing BMSCs under normoxic and hypoxic conditions, displayed that miR-328-3p expression was up-regulated in hypoxia-treated BMSC-EVs. And hypoxic BMSC-EVs could deliver the highly expressed miR-328-3p to lung cancer cells, thus facilitating the proliferation of lung cancer cells, and accelerating the growth of tumors *in vivo*. Further findings revealed that the tumor-promoting effect was achieved by restraining the Hippo pathway via targeting NF2 gene (Liu X. et al., 2021). What’s more, the hypoxic BMSC-derived exosomal miR-652-3p has a comparable impact on HCC (Li et al., 2023). Another miR-208a from BMSC-Exos also favored osteosarcoma cell proliferation (Qin et al., 2020).

In conclusion, miRNAs in MSC-EVs under varying conditions have diverse effects on tumor cell proliferation in different tumors (Table 1).

**TABLE 1:** Effects of miRNAs in MSC-EVs on tumor cell proliferation.

Tumors	Source of MSCs	microRNAs	Targets	Function	References
Colorectal cancer	Bone marrow	miR-99b-5p, miR-16-5p, miR-22-3p	FGFR3, ITGA2, RAP2B and PI3K/AKT Pathway	↓Proliferation	Xu Y. et al. (2019); Wang and Lin (2021); Ning et al. (2023)
	Umbilical cord	miR-1827	SUCNR1	↓Proliferation	Chen J. et al. (2023)
Cervical cancer	Bone marrow	miR-331-3p, miR-144-3p	DNMT3A, CEP55	↓Proliferation ↑Apoptosis	Meng et al. (2021); Yang S. et al. (2022)
Breast cancer	Bone marrow	miR-106a-5p	—	↑Proliferation ↑Cell viability	Xing et al. (2020)
	Adipose tissue	miR-381-3p	Wnt signaling pathway	↓Proliferation ↑Apoptosis	Shojaei et al. (2021)
	Dental pulp	miR-34a	—	↓Proliferation	Vakhshiteh et al. (2021)
Esophageal squamous cell carcinoma	Umbilical cord	miR-655-3p, miR-375	LMO4/HDAC2, ENAH	↓Proliferation	He et al. (2020); Chen M. et al. (2023)
Bladder cancer	Bone marrow	miR-139-5p	KIF3A/p21	↓Proliferation ↑Apoptosis	Xiang et al. (2022)
Prostate cancer	Bone marrow	miR-205	RHPN2	↓Proliferation ↑Apoptosis	Jiang et al. (2019)
Pancreatic ductal adenocarcinoma	Umbilical cord	miR-145-5p	—	↓Proliferation ↑Apoptosis	Ding et al. (2019)
Ovarian cancer	—	miR-18a-5p	NACC1/AKT/mTOR	↓Proliferation	Wang X. et al. (2022)
Thyroid-like cancer	Umbilical cord	miR-30c-5p	PELI1/PI3K/AKT	↓Proliferation	Zheng et al. (2022)
Lung cancer	Bone marrow	let-7i	KDM3A/DCLK1/FXYD3	↓Proliferation	Liu J. et al. (2021)
Nasopharyngeal carcinoma	—	miR-34c	β-Catenin	↓Proliferation ↑Apoptosis	Wan et al. (2020)
Hepatocellular carcinoma	Adipose tissue	miR-125b	—	↓Proliferation	Baldari et al. (2019)
Osteosarcoma	Bone marrow	miR-206	TRA2B	↓Proliferation ↑Apoptosis	Zhang H. et al. (2020)
Glioma	Bone marrow	miR-375, miRNA-199a	SLC31A1, AGAP2	↓Proliferation	Yu et al. (2019); Deng et al. (2020)
		miR-503	KIF5A	↑Proliferation	Wang X.-S. et al. (2022)
Wilms tumor	Umbilical cord	miR-15a-5p	SEPT2	↓Proliferation ↑Apoptosis	Huang et al. (2022)

### 3.2 Involvement in tumor invasion and metastasis

Activation of cancer cell invasion and metastasis is one of the main features of tumors (Hanahan and Weinberg, 2011). The formation of metastasis is the result of a complex multistep cascade process. The basic steps involve the detachment of cancer cells from the primary tumor, the occurrence of local invasion, and then the cells entering the circulatory system (intravasation) and extravasating to distant organs, eventually forming the secondary tumor (Yang et al., 2020; Dymerska and Marusiak, 2024). Numerous studies have shown that miRNAs in MSC-EVs have the capacity to regulate tumor cell migration and invasion, ECM remodeling, epithelial-mesenchymal transition (EMT), and distant metastasis as well. It is worth mentioning that angiogenesis is also a factor affecting tumor metastasis, which will be described separately elsewhere.

#### 3.2.1 Inhibition of tumor invasion and metastasis

MiRNAs in MSC-EVs can suppress the local invasion of tumors. For example, FXYD3 is a sodium-potassium ATPase regulator, which is a key mediator in multiple tumors. Let-7i derived from BMSC-EVs could reduce FXYD3 expression by inhibiting DCLK1 via KDM3A (Wang et al., 2017). Through this mechanism, let-7i declined the proliferation, migration, and invasion of lung cancer cells (Liu J. et al., 2021). BMSC-Exos could carry and translocate miR-206 to osteosarcoma cells, thereby attenuating cell migration and invasion by targeting TRA2B (Zhang H. et al., 2020). Another study reported that miR-375 was delivered to esophageal squamous cell carcinoma cells via hUCMSC-Exos. MiR-375 subsequently decreased the expression of ENAH and repressed the invasion and migration of esophageal squamous cell carcinoma (ESCC) cells by regulating the expression levels of EMT-related proteins (E-cadherin, N-cadherin and Snail) (He et al., 2020). Additionally, exosomes secreted by hBMSCs could also transfer miR-205 into prostate cancer cells and suppress the invasion and migration of tumor cells by restraining RHPN2 (Jiang et al., 2019). On a similar note, BMSC-Exos over-expressing miR-16-5p curtailed CRC progression by down-regulating ITGA2 (Xu Y. et al., 2019).

ECM is involved in the composition of the TME and plays a very critical role in altering the phenotypic and functional characteristics of tumor cells and stromal cells (Wu et al., 2019). MiRNAs in MSC-EVs can mediate ECM remodeling, thereby favoring the local invasion of tumor cells. For example, miR-331-3p repressed the expression of DNMT3A in cervical cancer cells by binding to DNMT3A mRNA. DNMT3A mediated the methylation of the CpG island at the promoter of LIMS2, and thus diminishing the expression of LIMS2 in cervical cancer cells. The LIMS2 gene encoded a protein (PINCH-2) that mediated the cytoskeletal and ECM interactions by forming a ternary complex (IPP complex) with ILK and parvin (Xu et al., 2016). Through the above mechanism, miR-331-3p provided by BMSC-EVs weakened the invasiveness of cervical cancer cells (Yang S. et al., 2022).

EMT is a cellular process, in which a phenotypic transformation of epithelial cells to mesenchymal cells occurs. EMT is activated during tumor pathogenesis, resulting in cells more migratory and often aggressive (Yang et al., 2020). Several studies have demonstrated that the regulatory effects of tumor invasion and metastasis by miRNAs in MSC-EVs are related to EMT. For instance, BMSC-derived exosomal miR-16-5p restrained EMT and hindered breast cancer progression by alleviating the activation of the NF-κB signaling pathway and down-regulating the expression of EPHA1 (Zhang Y. et al., 2022). MiR-34a-5p in MSC-EVs suppressed CRC cell growth and EMT process by influencing c-MYC binding to DNMT3a and epigenetically regulating PTEN (Zhao et al., 2022). Similarly, MSC-Exos repressed EMT, invasion, and migration of CRC cells through miR-100 and miR-143 (Jahangiri et al., 2022). Moreover, AMSC-Exos loaded with miR-381 reduced TNBC cell viability, migration, and invasiveness by targeting Wnt signaling pathway and EMT-related transcription factors (Shojaei et al., 2021). MiR-34c in MSC-Exos exhibited the similar tumor-suppressive impact on nasopharyngeal carcinoma via targeted inhibition of β-catenin (Wan et al., 2020).

MiRNAs in MSC-EVs can also modulate the distant metastasis of tumors. MiR-101-rich EVs from AMSCs could be taken up by osteosarcoma cells, and systemic injection of these EVs effectively inhibited tumor metastasis *in vivo* without obvious side effects (Zhang K. et al., 2020). One study found that miR-655-3p in hUCMSC-EVs suppressed the liver metastasis of ESCC by inactivating HIF-1α via the LMO4/HDAC2 axis (Chen M. et al., 2023). BMSC-EVs delivered miR-139-5p to bladder cancer cells, leading to the down-regulation of KIF3A and activation of p21. This process prevented the tumorigenesis and metastasis of bladder cancer cells *in vivo* (Xiang et al., 2022). Likewise, miR-1827 carried by hUCMSC-Exos could hamper the liver metastasis of CRC *in vivo* (Chen J. et al., 2023).

#### 3.2.2 Facilitation of tumor invasion and metastasis

Although most of the current studies focus on the inhibitory effects of miRNAs in MSC-EVs on tumor invasion and metastasis, they can also play a pro-metastatic role. Zhang et al. examined the expression profiles of exosomal miRNAs in BMSCs under hypoxic condition. And it was demonstrated that compared with normoxic BMSCs, the expression of some exosomal miRNAs in hypoxic BMSCs, including miR-146a-5p, miR-574-3p, miR-328-3p, miR-326-3p, miR-193a-3p, miR-5100, and miR-210-3p were up-regulated, while the other exosomal miRNAs, such as miR-6404, miR-6995-3p, and miR-5112 were down-regulated. Then they selected miR-193a, miR-210-3p, and miR-5100 for further study. The results revealed that the above miRNAs in hypoxic BMSC-Exos could activate STAT3 signaling-induced EMT, which gave rise to the enhanced invasive properties of lung cancer cells (Zhang et al., 2019). Another subsequent study confirmed that miR-328-3p induced by hypoxia could play a similar role through NF2 gene-mediated inhibition of the Hippo pathway (Liu X. et al., 2021). Besides, miR-652-3p in hypoxic BMSC-Exos contributed to HCC metastasis by targeting TNRC6A (Li et al., 2023). BMSC-derived exosomal miR-208a could boost the viability, migration, and clonogenicity of osteosarcoma cells (Qin et al., 2020).

Overall, miRNAs in MSC-EVs possess the ability to manipulate the local invasion and distant metastasis of tumors by interfering with multiple steps of tumor metastasis (Table 2).

**TABLE 2:** Effects of miRNAs in MSC-EVs on tumor invasion and metastasis.

Tumors	Source of MSCs	microRNAs	Targets	Function	References
Colorectal cancer	Bone marrow	miR-99b-5p, miR-22-3p	FGFR3, RAP2B and PI3K/AKT Pathway	↓Invasion	Wang and Lin (2021); Ning et al. (2023)
	Umbilical cord	miR-1827	SUCNR1	↓Migration ↓Invasion ↓Metastasis	Chen J. et al. (2023)
Breast cancer	Bone marrow	miR-106a-5p	—	↑Migration ↑Invasion	Xing et al. (2020)
		miRNA-551b-3p	TRIM31/Akt	↓Migration	Yang Z. et al. (2022)
	Umbilical cord	miR-3182	mTOR and S6KB1 genes	↓Migration	Khazaei-Poul et al. (2021)
	Adipose tissue	miR-16-5p	—	↓Migration	Li et al. (2020)
Bladder cancer	Bone marrow	miR-139-5p	KIF3A/p21	↓Migration ↓Invasion ↓Metastasis	Xiang et al. (2022)
	Bladder	miR-217	Hippo-YAP	↑Migration	Huang et al. (2021)
Ovarian cancer	—	miR-18a-5p	NACC1/AKT/mTOR	↓Migration ↓Invasion	Wang X. et al. (2022)
Cervical carcinoma	Bone marrow	miR-144-3p	CEP55	↓Migration ↓Invasion	Meng et al. (2021)
Thyroid-like cancer	Umbilical cord	miR-30c-5p	PELI1/PI3K/AKT	↓Migration	Zheng et al. (2022)
Mammary tumor	Bone marrow	let-7f	—	↓Invasion	Egea et al. (2021)
Pancreatic ductal adenocarcinoma	Umbilical cord	miR-145-5p	—	↓Invasion	Ding et al. (2019)
Lung cancer	Bone marrow	miR-21-5p	—	↑Mobility ↑EMT	Ren et al. (2019)
Esophageal cancer	Umbilical cord	miR-655-3p	LMO4/HDAC2	↓Metastasis	Chen M. et al. (2023)
Hepatocarcinoma cancer	Bone marrow	miR-652-3p	TNRC6A	↑Metastasis	Li et al. (2023)
Osteosarcoma	Adipose tissue	miR-101	BCL6	↓Migration ↓Invasion ↓Metastasis	Zhang K. et al. (2020)
	—	miRNA-22	Twist1/CADM1	↓Migration ↓Invasion	Ruan et al. (2024)
Glioma	Bone marrow	miR-375, miR-199a, miR-133b	SLC31A1, AGAP2, EZH2	↓Migration ↓Invasion	Xu H. et al. (2019); Yu et al. (2019); Deng et al. (2020)
		miR-29a-3p	—	↓Migration	Zhang et al. (2021)
		miR-503	KIF5A	↑Migration ↑Invasion	Wang X.-S. et al. (2022)
Wilms tumor	Umbilical cord	miR-15a-5p	SEPT2	↓Migration ↓Invasion	Huang et al. (2022)

### 3.3 Participation in immunomodulation

Tumor progression is also influenced by immunomodulation. MiRNAs in MSC-EVs positively or negatively regulate immune function by affecting various pathways. It is reported that miR-503 in MSC-EVs directly targeted KIF5A, which facilitated the release of immunosuppressive factors through IL-7 signaling pathway. Such mechanism enhanced tumor cell proliferation, migration and invasion, as well as immune escape, and attenuating T-cell proliferation in glioma cells (Wang X.-S. et al., 2022). In addition, miR-222 in MSC-EVs could modulate AKT1 transcription in CRC by interacting with ATF3, thereby propelling immune escape from CRC cells (Li et al., 2021).

As an important component of the TME, M2 polarization of tumor-associated macrophages (TAMs) provides an immunosuppressive niche for tumor progression (Yang et al., 2015). MiR-744-5p in MSC-EVs could relieve MAPK signaling activity through down-regulating TGFB1, which in turn reduced macrophage M2 polarization and thus prevented glioma progression (Liu L. et al., 2022). Similarly, exosomes from hUCMSCs carrying miR-1827 attenuated M2 macrophage polarization through SUCNR1 down-regulation, thereby restraining CRC cell migration and invasion (Chen J. et al., 2023).

### 3.4 Influence on angiogenesis

Pathological angiogenesis is one of the hallmarks of cancer and also an essential process in tumor growth and metastasis (Carmeliet and Jain, 2000). MSC-Exos with high expression of miR-29a-3p declined angiogenic mimicry formation in glioma cells, which was an alternative microvascular cycle independent of vascular endothelial growth factor (VEGF)-driven angiogenesis (Zhang et al., 2021). MiR-100-5p and miR-1246 enriched in exosomes derived from human dental pulp stem cells could target VEGFA in endothelial cells. This process refrained endothelial cell proliferation and migration, and induced cell apoptosis, ultimately alleviating angiogenesis and exerting an anti-tumor effect on oral squamous cell carcinoma (Liu P. et al., 2022).

### 3.5 Impact on chemotherapy sensitivity

Chemotherapy is an important strategy for tumor treatment, however, drug resistance of tumor cells restricts its application and development (Lohitesh et al., 2018). The effects of miRNAs on drug resistance in tumors have long been recognized and systematically reviewed (Sarkar et al., 2010). In recent years, researchers have attempted to intervene in this situation with miRNAs in MSC-EVs. For e.g., Wu et al. found that miR-193a was less expressed in cisplatin (DDP)-resistant tissues compared with DDP-sensitive tissues, suggesting that miR-193a might be associated with DDP resistance. Subsequently, NSCLC DDP-resistant cells and mice were treated with BMSC-Exos, which were highly expressed miR-193a. And it was demonstrated that BMSC-Exos restrained the proliferation and augmented the apoptosis of NSCLC cells *in vitro*, and dampened the growth of NSCLC graft tumors in mice *in vivo*. Further, this inhibitory effect was achieved by down-regulating LRRC1 (Wu et al., 2020). Another study reported that miR-301b-3p expression was elevated in cisplatin- and vincristine-resistant gastric cancer tissues, whereas MSC-EVs could down-regulate TXNIP by delivering miR-301b-3p, which induced drug resistance and aggravated malignant behaviors of gastric cancer (GC) cells *in vitro* and *vivo* (Zhu et al., 2023).

Many studies have modified MSCs to explore the possibility of delivering miRNAs via EVs to improve drug resistance in tumors. Lou et al. demonstrated that transfection of miR-122 in AMSCs, a miRNA that has been shown to be associated with HCC chemosensitivity (Bai et al., 2009), could effectively package miR-122 into secreted exosomes. These exosomes could mediate miR-122 communication between AMSCs and HCC cells, thereby sensitizing cancer cells to chemotherapeutic agents by altering the expression of miR-122 target genes in HCC cells. Moreover, intratumoral injection of miR-122-rich Exo remarkably increased the anti-tumor effect of sorafenib against HCC *in vivo* (Lou et al., 2015). Temozolomide (TMZ) chemotherapy is one of the main treatments for glioma (Corso et al., 2017), MSC-Exos over-expressing miR-199a could augment the chemosensitivity of glioma cells to TMZ and retard tumor growth *in vivo* (Yu et al., 2019). Another comparable study found that AMSC-derived miR-199a-modified exosomes efficiently mediated the delivery of miR-199a to HCC cells, thus enhancing the chemosensitivity of HCC cells to doxorubicin *in vivo* and *in vitro* through mTOR pathway inhibition (Lou et al., 2020). Additionally, exosomal miR-146a derived from hUCMSCs elevated the sensitivity of OC cells to docetaxel and PTX via LAMC2-mediated PI3K/Akt axis (Qiu et al., 2020). MiR-21-5p strengthened the breast cancer drug resistance in the MSC-secreted exosome by up-regulating S100A6 expression (Luo et al., 2020). Another study reported that hMSC-EV-derived miR-18a-5p declined OC cell proliferation, migration and invasion, and ameliorated cell sensitivity to DDP. Although miR-18a-5p hindered OC progression by targeting the NACC1/AKT/mTOR axis, it was not clear whether this axis was correlated with miR-18a-5p-mediated chemosensitivity (Wang X. et al., 2022).

**FIGURE 2:**
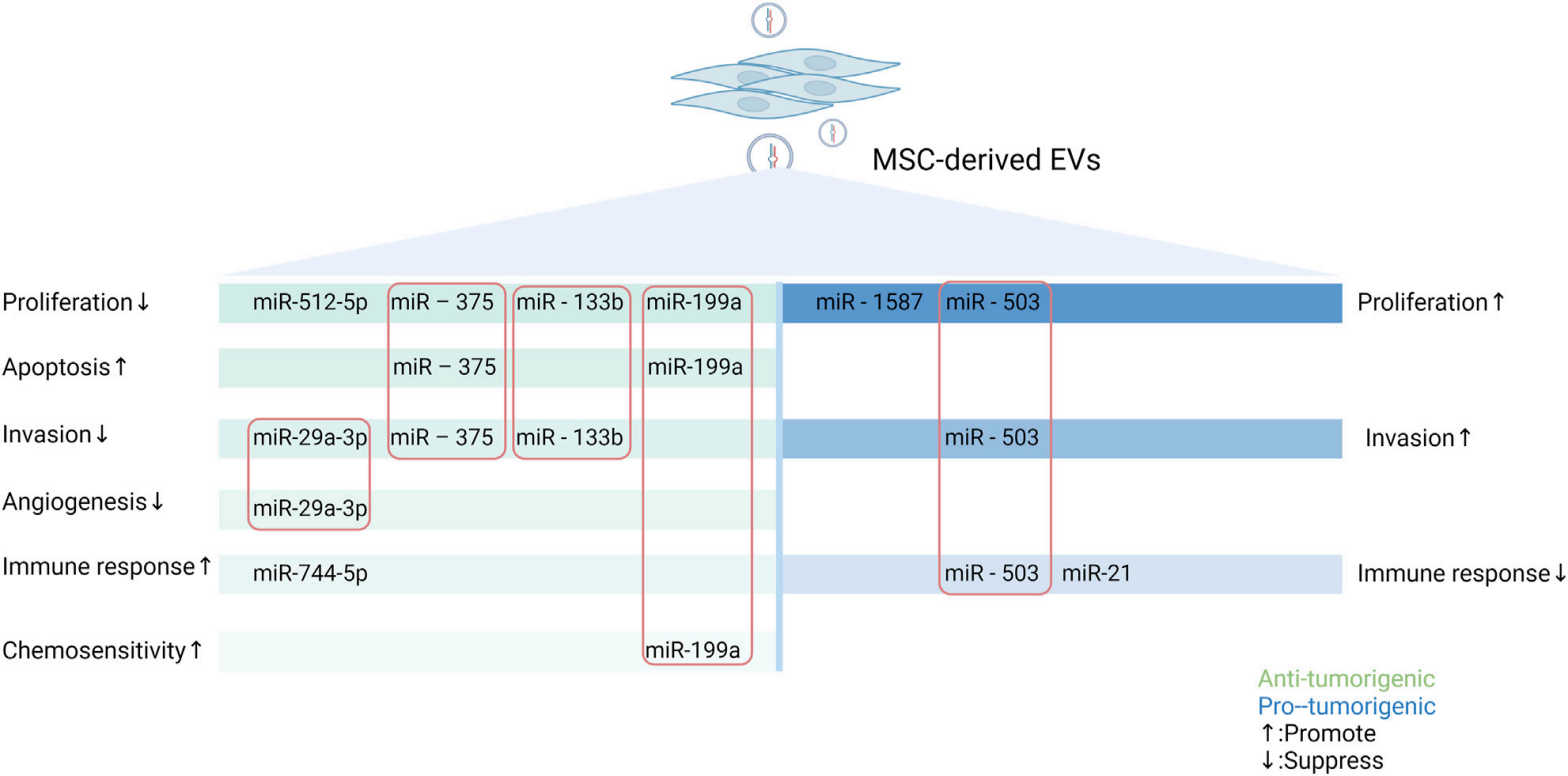
Schematic representation of the pro- and anti-tumor effects of miRNAs in MSC-EVs in gliomas through multiple mechanisms. MSCs can secrete miRNAs-containing EVs to regulate the biological behaviors of tumor cells, thus exerting different impacts on tumor. MiRNAs in MSC-EVs can promote or inhibit tumorigenesis by influencing tumor cell proliferation and apoptosis, altering tumor cell invasion and metastasis, mediating immune regulation, modulating angiogenesis, and modifying the expression of drug-resistant genes. In this figure, green represents tumor-promoting properties and blue represents anti-tumor properties. The miRNAs enclosed in the rectangle are those that have important roles in the process of tumorigenesis (Created with Biorender.com).

In conclusion, EV-miRNAs from MSCs promote or inhibit tumor progression by regulating tumor cell proliferation and apoptosis, invasion and metastasis, mediating immune modulation, and influencing angiogenesis as well as chemosensitivity. In addition, other mechanisms intervening in tumor progression have also been reported, such as interference with mitochondrial metabolism (Lin et al., 2023) and radioresistance (Wan et al., 2020). Notably, the above mechanisms do not play a single role but affect multiple aspects of the tumors. For example, EVs secreted by hypoxic pre-stimulated MSCs delivered miR-21-5p into NSCLC cells, which not only accelerated tumor growth *in vivo* but also facilitated intratumoral angiogenesis and macrophage M2 polarization by decreasing the expression of PTEN, PDCD4 and RECK genes in NSCLC cells (Ren et al., 2019). The various pro- and anti-tumor effects and mechanism of miRNAs in MSC-EVs are demonstrated in Figure 2 using the more studied glioma as an example.

## 4 Function of miRNAs in EVs in the crosstalk between MSCs and tumor cells

**FIGURE 3:**
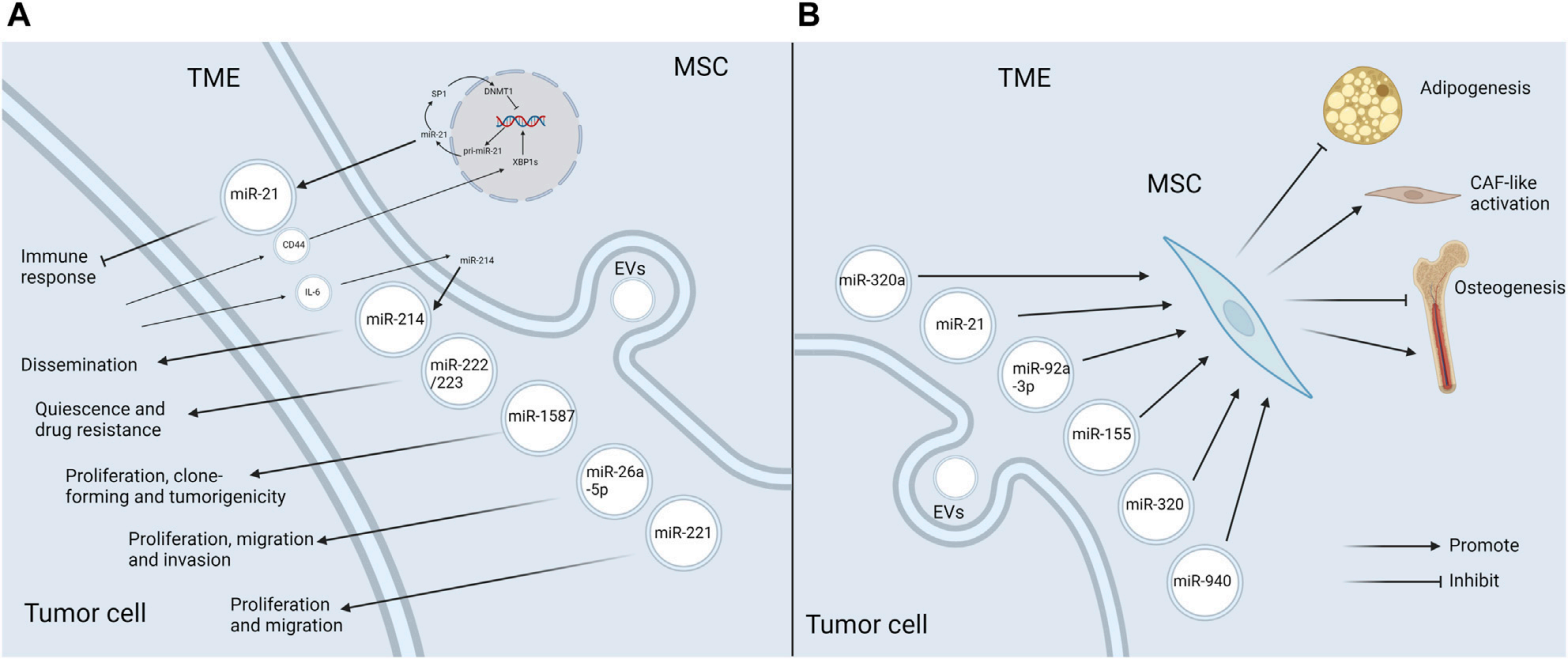
EV-miRNAs mediate the crosstalk between MSCs and tumor cells in the TME **(A)** MSCs release specific EV-miRNAs to boost tumor progression in the TME. MSCs in TME secrete EVs enriched in miR-21, miR-214, miR-222/223, miR-1587, miR-26a-5p, and miR-221, which favor tumor progression through the diverse mechanisms. Additionally, the release of miR-21 and miR-214 is also influenced by the tumor cells. CD44 in glioma exosomes triggers the miR-21/SP1/DNMT1 positive feedback loop in MSCs, which causes GA-MSCs to secrete miR-21-rich exosomes, thus having an immunosuppressive effect. Tumor cells induce MSCs to produce miR-214-rich EVs upon the activation of IL-6/STAT3 signaling, which can facilitate tumor proliferation. **(B)** Tumor cells affect MSCs in the TME by releasing EV-miRNAs. MiR-92a-3p and miR-155 attenuate adipogenesis in ADSCs. MiR-320 inhibits osteogenesis in BMSCs, whereas miR-940 promotes osteogenesis. Besides, miR-320a and miR-21 induce the transformation of MSCs into CAFs (Created with Biorender.com).

During the process of tumor progression, tumor cells constantly communicate with the components in TME, and EVs and miRNAs play a special role (Bakhsh et al., 2022). This paper focuses on the function of miRNAs in EVs in the crosstalk between MSCs and tumor cells. Tumor cells recruit MSCs to the TME, alter their functional characteristics, and further reprogramm them into tumor-associated MSCs, which in turn have an impact on tumor progression (Whiteside, 2018). It has been shown that when MSCs are recruited to the sites of liver injury, they acquire a cancer-promoting phenotype under the influence of the TME, which is partly mediated by dysregulated expression of intracellular miRNAs (Salah et al., 2022). In addition, the biological behaviors of MSCs in TME can also be modulated by EV-miRNAs secreted by tumor cells. Altogether, miRNAs, as mediators of the interplay between tumor cells and MSCs, exert the key roles in tumor progression (Figure 3).

### 4.1 MSCs in TME affect tumor cells through EV-miRNAs

MSCs in TME exhibit a different secretome profile from normal tissue MSCs, which favors tumor progression. Research suggested that the levels of certain miRNAs in EVs derived from gastric cancer tissue MSCs were significantly discrepant compared with the neighboring noncancerous tissue MSCs. Among them, miR-221, which was markedly up-regulated in gastric cancer tissue MSCs, could be delivered to gastric cancer cells via exosomes and accelerate cell proliferation and migration (Wang et al., 2014). One study reported that miR-26a-5p was highly expressed in small-sized EVs from BMSCs of AML patients compared with healthy controls. MiR-26a-5p could diminish GSK3β expression and activate Wnt/β-catenin signaling pathway in AML cells, thus promoting AML progression (Ji et al., 2021). In another study, specific miRNAs were found to be enriched in exosomes derived from glioma-associated MSCs (GA-MSCs). Among these miRNAs, miR-1587 could target the tumor suppressor NCOR1 in glioma Stem-like Cells and enhance their proliferative and clone-forming capacity *in vitro*, as well as the tumorigenicity *in vivo* (Figueroa et al., 2017).

Importantly, the alterations of miRNAs in MSC-EVs in TME and the corresponding promoting effects may be the result of tumor pathogenesis. It has been reported that breast cancer cells could stimulate MSCs to release exosomes containing diverse miRNAs, such as miR-222/223, which in turn induced quiescence and drug resistance in a subset of cancer cells (Bliss et al., 2016). Another study reached a consistent conclusion by establishing the co-culture of hBMSCs with osteosarcoma cells (Qi et al., 2021). Some specific mechanisms have been explored. Qiu et al. demonstrated that CD44 in glioma exosomes could trigger a miR-21/SP1/DNMT1 positive feedback loop in MSCs, which led to the secretion of miR-21-rich exosomes by GA-MSCs. Then exosomal miR-21 further contributed to the progression of gliomas by immunosuppression. Interestingly, the immunosuppressive mechanism induced by exosomal miR-21 secreted by GA-MSCs was similar to, but more intense than, the miR-21-mediated immunosuppressive signaling in glioma exosomes. Thereby, this study reveals the critical role of MSCs as a signal multiplier in the glioma microenvironment to enhance immunosuppressive signaling of glioma exosomes (Qiu et al., 2023). Another study showed that tumor cells were able to induce stromal cells, including MSCs, to produce miR-214-rich EVs upon the activation of IL-6/STAT3 signaling, which favored the tumor dissemination (Orso et al., 2023). To sum up, MSCs in TME can facilitate tumor progression via releasing pro-oncogenic EV-miRNAs, and the production of specific EV-miRNAs can also be influenced by tumor cells (Figure 3A).

### 4.2 Tumor cells influence MSCs in TME via EV-miRNAs

Tumor cells can also have an impact on MSCs in TME via EV-miRNAs. A good example of this event is miR-92a-3p. MiR-92a-3p is over-expressed in the exosomes of a variety of cancer cells including chronic myelogenous leukemia (CML). In CML, it has been demonstrated that miR-92a-3p could be transported to ADSCs and slow down their adipogenesis by repressing the adipose promoter C/EBPα, thus contributing to cancer-associated malignant stroma (CAC) formation (Wan et al., 2019). It ultimately led to a systemic syndrome characterized by weight loss, skeletal muscle atrophy and adipose tissue atrophy (Petruzzelli and Wagner, 2016). Similarly, GC exosomal miR-155 was shown to target C/EPBβ in AMSCs, which in turn suppressed adipogenesis and enhanced brown adipose differentiation, thereby correlating with CAC formation (Liu Y. et al., 2022). In addition, CML cells could selectively sort some tumor suppressor miRNAs, such as miR-320, into exosomes via HNRNPA1, an RNA-binding protein. These exosomes would be endocytosed by neighboring BMSCs and subsequently attenuate their osteogenesis, thereby remodeling the bone marrow niche that favored CML progression. Intriguingly, this selective sorting of miRNAs also directly accelerated cell growth in CML (Gao et al., 2019). Conversely, the exosome miR-940 from prostate cancer cells facilitated hMSC osteogenesis *in vitro* and induced the extensive osteogenic lesions in the bone metastatic microenvironment *in vivo* (Hashimoto et al., 2018). Furthermore, cancer-associated MSCs in TME could differentiate into CAFs, which boosted tumor progression (Borriello et al., 2017). MiRNAs in EVs secreted by tumor cells can induce this transformation process. For example, miR-320a in small EVs secreted by tumor cells targeted ITGA7 to activate TGF-β pathway, which drove CAF-like activation of ADSCs and thus facilitated omental metastasis of OC (Gong et al., 2024). Likewise, exosomes from head and neck squamous cell carcinoma cells could trigger CAFs-like features in hBMSCs, which was mediated by miR-21 (Wang et al., 2023). The above studies indicate that tumor cells can produce EV-miRNAs to act on MSCs, causing them to develop in a direction conducive to tumor progression (Figure 3B).

Taken together, tumor cells and MSCs create a niche supporting tumor progression or regression via the complex interaction of EV-miRNAs in TME.

## 5 Applications of miRNAs in MSC-EVs in tumor pharmacotherapy

### 5.1 Engineering modifications to EVs for drug delivery

MSC-EVs have the unique advantages compared with EVs derived from bodily fluids, other cellular sources, and other nanocarriers in terms of drug delivery (Weng et al., 2021). There are two main strategies for introducing miRNAs into MSC-EVs. One is *in vivo* drug loading. The genes to be studied are transferred into MSCs by gene transfection methods, and then EVs loaded with the target miRNAs are extracted and purified. Lentiviral transduction is one of the more commonly used strategies. The other is *in vitro* drug loading. MiRNAs were loaded into purified EVs by using liposome fusion (Li et al., 2020) and other techniques (Lai et al., 2013).

Targeting ability is an issue often considered when utilizing EVs as drug carriers. The cellular origins of EVs notably influences their preferential homing sites (Herrmann et al., 2021). To ameliorate the targeting specificity of EVs, surface engineering is a useful tool (Liang et al., 2021). In a recent study, natural free streptavidin (SA) was genetically engineered on the cell surface of BMSCs to obtain SA-expressing BMSC-EVs. Based on the high affinity for biotinylated molecules, SA can be paired with a variety of biotinylated molecules and thus be endowed with various targeting properties. Subsequently, researchers modified SA-EVs with different biotinylations and demonstrated the feasibility of SA-EVs for targeted drug delivery in a variety of situations (Meng et al., 2023). Another study constructed an exosome over-expressing the fusion proteins Lamp2b-IL3 and HCELL, and verified that the engineered exosomes could enhance bone marrow homing and selective targeting of leukemia stem cells. After loading miR-34c-5p into the engineered exosomes, the ability of target tumor suppression was further confirmed in an AML mouse model (Wen et al., 2023).

EVs are often used as an ideal natural endogenous nanodelivery system for drug delivery due to their unique origin, structure and physiological functions (Banerjee and Rajeswari, 2023). Furthermore, EVs can encapsulate synthesized nanoparticles in membranes to obtain biomimetic nanoparticles, which have both the advantages of synthesized nanoparticles and the properties of EVs source cells (Liu et al., 2023). A study demonstrated that mesoporous silica nanoparticles were encapsulated into an exosome to efficiently deliver drugs to cancer cells (Sarkar et al., 2024). Xu et al. constructed gold-coated magnetic nanoparticles, and then applied these nanoparticles to load EVs, augmenting EVs-based drug delivery (Xu et al., 2023).

### 5.2 Clinical applications of EVs for cancer therapy

**FIGURE 4:**
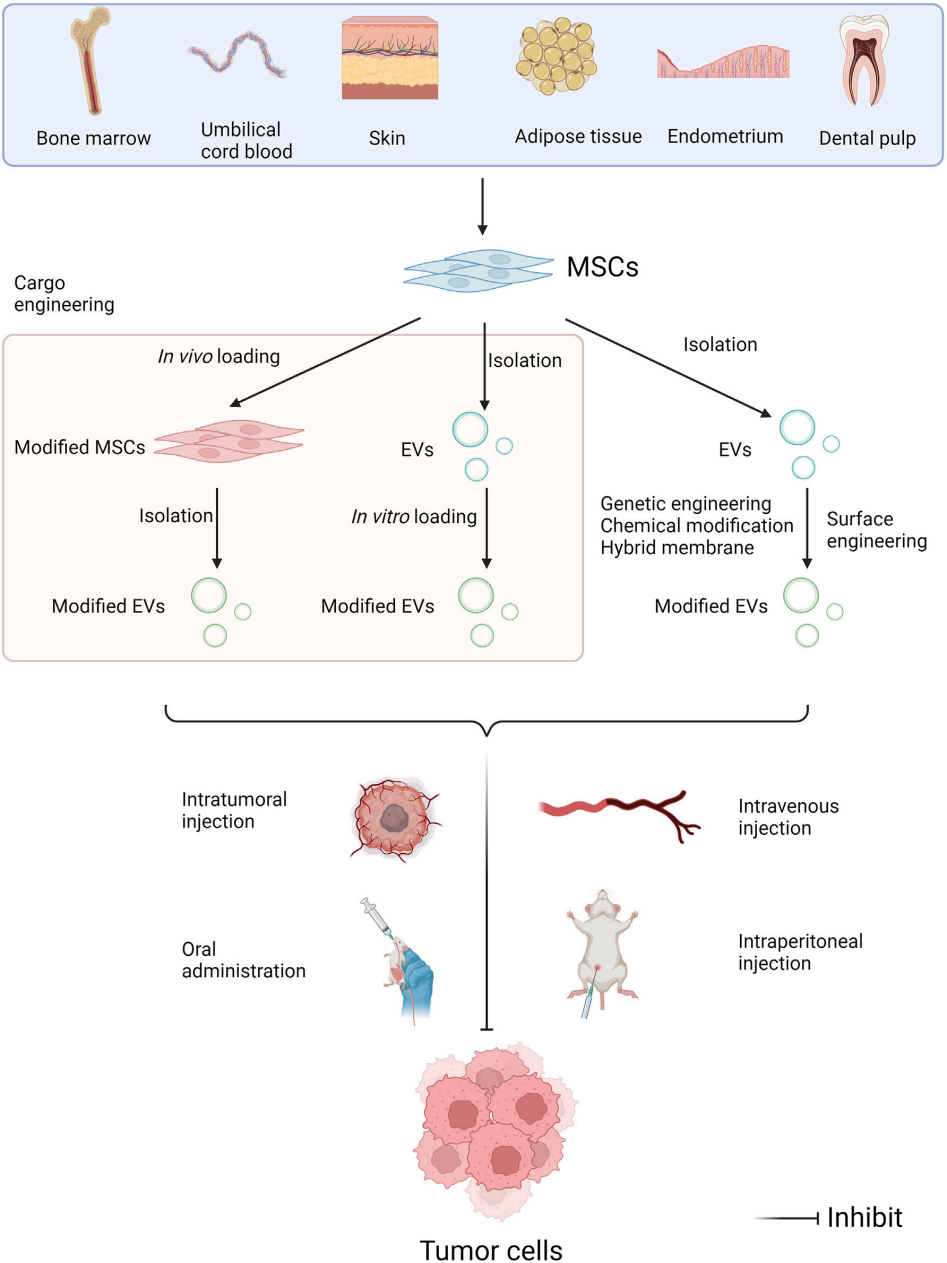
Administration approaches of miRNAs in MSC-EVs in tumor pharmacotherapy. MSCs can be isolated from multiple human tissues such as bone marrow, umbilical cord blood, skin, adipose tissue, endometrium, dental pulp, and so on. EV modifications include cargo engineering and surface engineering. Cargo engineering enables miRNAs to be transferred into MSC-EVs, including *in vivo* loading and *in vitro* loading. Surface engineering can enhance the target specificity of EVs, including genetic engineering, chemical modification, and hybridized membrane. Then EVs can be administrated intravenously, intratumorally and intraperitoneally, as well as orally, further exerting the anti-tumor effects (Created with Biorender.com).

As previously mentioned, the utility of MSC-EVs loaded with anti-tumor miRNAs or inhibitors of pro-cancer miRNAs to repress tumor progression has a well-established theoretical basis and has yielded many results in preclinical studies. The applications of MSC-EVs in cancer therapy mainly involve the drug delivery as a vehicle, and a direct therapeutic modality through their miRNA cargoes (Abdulmalek et al., 2024). Currently, the main routes of EVs applications include intravenous, intratumoral and intraperitoneal injection, as well as oral administration (Figure 4). Multiple studies have indicated that exosomes modified with targeted ligands can be effectively used to deliver chemotherapeutic agents to tumors via intravenous injection (Ding et al., 2019). So far, MSC-EVs have already under the clinical assessment for future applications in cancer treatment. An ongoing phase I clinical study has been exploring the therapeutic effectiveness of MSC-Exos with KrasG12D siRNA in the patients with metastatic pancreas cancer carrying KrasG12D mutation (NCT03608631). Another study validates the safety and efficacy of UCMSC-Exos in promoting recovery of chemotherapy-induced myelosuppression in patients with AML (NCT06245746).

## 6 Conclusion and prospects

There is no doubt that miRNAs carried by MSC-EVs are involved in tumor pathogenesis in various ways and play a vital role in tumor progression. A large number of related studies have demonstrated their potential significance for clinical applications. This article reviewed the multiple roles and mechanism, by which MSC-derived EV-miRNAs influence tumor progression, the function of EV-miRNAs in the interaction between tumor cells and MSCs in TME, and how to modify MSC-derived EV-miRNAs to facilitate their clinical applications (Graphical Abstract).

Despite the promising clinical applications of miRNAs in MSC-EVs, there are still many urgent problems to be solved in tumor research and applications. First, the types of MSCs used in the studies are not uniform. Previous studies have shown that the immune properties of different species or subpopulations of MSCs (Shi et al., 2018), the ability to migrate *in vivo* and transplant to tumors (Belmar-Lopez et al., 2013), the effects on tumors (Li et al., 2020), and other characteristics are not consistent. Therefore, the types of EV-miRNAs derived from different species of MSCs and their impacts on tumors may also be diverse. Correspondingly, the inhibitory effects of the same miRNAs derived from MSC-EVs on different strains of tumor cells are also variable. It has been reported that the miR-124a delivered by MSC-Exos, an anti-glioma miRNA, exhibited varying inhibitory effects on the growth of different glioma stem cell strains. Other anti-glioma miRNAs also displayed selective inhibitory influences on glioma stem cell in different strains (Lang et al., 2018). In addition, many other bioactive substances besides miRNAs are present in EVs, which may affect the regulatory roles of miRNAs or have a direct impact on tumor progression. Although there is no very conclusive evidence, it has been indicated that other factors rather than miRNAs in MSC-EVs are also involved in the pro-cancer and immunomodulation of EVs (Ren et al., 2019). Ultimately, since each miRNA can potentially regulate hundreds of mRNAs (Kong et al., 2012), off-target issues should also be taken into account. As a result, future studies on the effects of miRNAs in MSC-EVs on tumor progression should be more refined to elucidate the influence of the above factors, such as the types of MSCs and different strains of tumor cells. More attention needs to be paid to the possible impact of the complex regulatory network among MSC-EVs, miRNAs and tumor cells on the basic research and clinical applications.

## Figures and Tables

**Table:** 

**AGAP2**	Ankyrin repeat and PH domain 2
**AML**	Acute myeloid leukemia
**AMSC**	Adipose mesenchymal stem cell
**BCL6**	B cell lymphoma 6
**BMSCs**	Bone marrow mesenchymal stem cells
**CAC**	Cancer-associated malignant stroma
**CAFs**	Cancer-associated fibroblasts
**CAE**	Colorectal cancer
**CCNE1**	Cell cycle protein E1
**CCNE2**	Cell cycle protein E2
**CEP55**	Centrosomal protein, 55 Kd
**CML**	Chronic myelogenous leukemia
**CRC**	Colorectal cancer
**DCLK1**	Doublecortin-like kinase 1
**DNMT1**	DNA methyltransferase 1
**DNMT3A**	DNA methyltransferase 3 alpha
**DDP**	Cisplatin
**ECM**	Extracellular matrix
**EMT**	Epithelial-mesenchymal transition
**ENAH**	Enabled homolog
**EPHA1**	Erythropoietin-producing hepatocellular A1
**ESCC**	Esophageal squamous cell carcinoma
**EVs**	Extracellular vesicles
**EV-miRNAs**	MiRNAs in EVs
**E2F2**	E2F family of transcription factor 2
**EZH2**	Enhancer of Zeste 2
**FGFR3**	Fibroblast growth factor receptor 3
**FOXA2**	Forkhead box A2
**FXYD3**	FXYD domain-containing ion transport regulator 3
**GA-MSCs**	Glioma-associated mesenchymal stem cells
**GC**	Gastric cancer
**HCC**	Hepatocellular carcinoma
**HDAC2**	Histone deacetylase 2
**HNRNPA1**	Heterogeneous nuclear ribonucleoprotein A1
**IGF1R**	Insulin-like growth factor 1 receptor
**ILK**	Integrin-linked kinase
**ITGA2**	Integrin α2
**ITGA7**	Integrin subunit alpha 7
**JAG1**	Jagged 1
**KDM3A**	Lysine demethylase 3A
**KIF3A**	Kinesin family member 3a
**KIF5A**	Kinesin family member 5A
**LAMP2B-IL3**	Lysosome-associated membrane protein 2-interleukin 3
**LAMC2**	Laminin γ2
**LMO4**	LIM-only protein 4
**LRRC1**	Leucine-rich repeat-containing protein 1
**LIMS2**	LIM zinc finger domain containing 2
**MELK**	Maternal embryonic leucine zipper kinase
**miRNAs**	MicroRNAs
**MVs**	Microvesicles
**MSCs**	Mesenchymal stem/stromal cells
**MSC-EVs**	MSC-derived EVs
**MSC-Exos**	MSC-derived exosomes
**NCOR1**	Nuclear receptor co-repressor
**NACC1**	Nucleus accumbens-associated protein 1
**NF-κB**	Nuclear factor-κB
**NF2**	Neurofibromin 2
**NFATc1**	Nuclear factor of activated T cells c1
**NSCLC**	Non-small cell lung cancer
**OSBPL11**	Oxysterol binding protein like 11
**OC**	Ovarian cancer
**PD-L1**	Programmed cell death ligand-1
**PELI1**	Pellino-1
**PI3K**	Phosphatidylinositol 3-kinase
**PTEN**	Phosphatase and tensin homolog
**PTX**	Paclitaxel
**RAP2B**	Rhoptry associated protein (RAP) 2B
**RHPN2**	Rhophilin Rho GTPase binding protein 2
**SEPT2**	Septin 2
**SLC31A1**	Solute carrier family 31 member 1
**SUCNR1**	Succinate receptor 1
**STAT3**	Signal transducers and activators of transcription 3
**SA**	Streptavidin
**TAMs**	Tumor-associated macrophages
**TMZ**	Temozolomide
**TNBC**	Triple-negative breast cancer
**TRA2B**	Transformer 2β
**TRIM31**	Tripartite motif-containing protein 31
**TME**	Tumor microenvironment
**TXNIP**	Thioredoxin interacting protein
**VEGF**	Vascular endothelial growth factor
